# Data Offloading in UAV-Assisted Multi-Access Edge Computing Systems: A Resource-Based Pricing and User Risk-Awareness Approach

**DOI:** 10.3390/s20082434

**Published:** 2020-04-24

**Authors:** Giorgos Mitsis, Eirini Eleni Tsiropoulou, Symeon Papavassiliou

**Affiliations:** 1 School of Electrical and Computer Engineering, National Technical University of Athens, 15780 Athina, Greece; gmitsis@netmode.ntua.gr (G.M.); papavass@mail.ntua.gr (S.P.); 2 Department of Electrical and Computer Engineering, University of New Mexico, Albuquerque, NM 87131, USA

**Keywords:** data offloading, UAV-enabled computing, resource-based pricing, risk-awareness, multi-access edge computing systems

## Abstract

Unmanned Aerial Vehicle (UAV)-assisted Multi-access Edge Computing (MEC) systems have emerged recently as a flexible and dynamic computing environment, providing task offloading service to the users. In order for such a paradigm to be viable, the operator of a UAV-mounted MEC server should enjoy some form of profit by offering its computing capabilities to the end users. To deal with this issue in this paper, we apply a usage-based pricing policy for allowing the exploitation of the servers’ computing resources. The proposed pricing mechanism implicitly introduces a more social behavior to the users with respect to competing for the UAV-mounted MEC servers’ computation resources. In order to properly model the users’ risk-aware behavior within the overall data offloading decision-making process the principles of Prospect Theory are adopted, while the exploitation of the available computation resources is considered based on the theory of the Tragedy of the Commons. Initially, the user’s prospect-theoretic utility function is formulated by quantifying the user’s risk seeking and loss aversion behavior, while taking into account the pricing mechanism. Accordingly, the users’ pricing and risk-aware data offloading problem is formulated as a distributed maximization problem of each user’s expected prospect-theoretic utility function and addressed as a non-cooperative game among the users. The existence of a Pure Nash Equilibrium (PNE) for the formulated non-cooperative game is shown based on the theory of submodular games. An iterative and distributed algorithm is introduced which converges to the PNE, following the learning rule of the best response dynamics. The performance evaluation of the proposed approach is achieved via modeling and simulation, and detailed numerical results are presented highlighting its key operation features and benefits.

## 1. Introduction

Towards realizing the emerging applications supported by the fifth generation (5G) wireless networks and the Internet of Things (IoT), while demanding ultra-reliable and low latency communication (URLLC), ubiquitous, distributed, and intelligent computing is one of the key enabling technologies. IoT is foreseen to reach 500 billion devices connected to the Internet by 2030 [1], while the global mobile traffic is expected to increase seven-fold by 2021 [2]. Thus, it is evident that traditional cloud computing architectures cannot support the latency constraints of the next generation networking environments, such as Tactile Internet [3]. The reasons are that the powerful cloud centers are often deployed far away from the end users; thus, huge amounts of traffic are usually transmitted through intermediate nodes resulting in heavy load, congestion, delay uncertainties, high energy consumption, and multiple security threats [4]. Thus, multi-access edge computing (MEC), which brings computing resources from the core network to the edge network, becomes a natural and promising solution to support these applications.

Combined with the MEC concept, Unmanned Aerial Vehicles (UAVs), equipped with communication and computing facilities, become a core component of next generation networks due to their salient attributes, such as hovering ability, flexibility and effortless deployment, maneuverability, mobility, low cost, strong line-of-sight (LoS) connection links, adjustable usage, and adaptive altitude [5]. The MEC servers are embedded in the UAVs that fly in closer proximity to the users compared to the conventional MEC servers typically residing at the Macro Base Stations (MBSs) of the macrocells or at the Access Points (APs) of the small cells [6]. Thus, the UAV-mounted MEC servers more efficiently support the end users applications’ data offloading and processing at the flying edge servers, by creating a flexible and dynamic computing environment paradigm [7].

### 1.1. Related Work

Cheng et al. [8] discuss the benefits introduced by the UAV-mounted MEC servers with respect to caching and computing, in a hybrid architecture consisting of UAV-mounted and ground MEC servers. Luo et al. [9] introduce a cloud-based UAV-assisted system and study its stability with respect to the sensors big data offloading rate. Valentino et al. [10] consider a fleet of UAV-mounted MEC servers and the optimization problem of increasing the UAVs fleet lifetime, while decreasing the overall computation time of the users’ offloaded tasks is formulated and solved. In particular, the authors exploit neighboring UAV clusters with sufficient computing resources to offload the users’ computation tasks.

Xiong et al. [11] formulate a joint optimization problem to optimize the users’ data offloading to the UAV-mounted MEC servers, the UAVs’ trajectory, and the data allocation during transmission to the different UAVs. An end-to-end solution is introduced by Jeong et al. [12], where the authors jointly optimize the users’ data offloading to the UAV-mounted MEC servers (i.e., uplink) and the output processed data returned to the users (i.e., downlink), while considering the computation tasks’ latency constraints. Jeong et al. [13] focus on the UAV-mounted MEC servers’ energy constraints to jointly optimize the users’ data offloading by considering orthogonal and non-orthogonal communication multiple access techniques, and the UAVs’ trajectory. Furthermore, Zhou et al. consider a wireless powered communication environment in [14,15], where the UAVs except from acting as UAV-mounted MEC servers providing computing services to the end-users, they also provide energy to them. Accordingly, the users can exploit the harvested energy to perform local computing and/or transmit their data to the UAV-mounted MEC servers.

### 1.2. Motivation and Contributions

All the aforementioned research works, though having demonstrated significant benefits and potential, have made two fundamental assumptions regarding the examined UAV-assisted multi-access edge computing system: (i) the UAV-mounted MEC servers offer their communication and computing services to the users for free; and (ii) the users act as neutral utility maximizers aiming at simply maximizing their perceived satisfaction from offloading and processing their data to the UAV-mounted MEC servers, thus exhibiting a risk-neutral behavior. However, in a realistic communication and computing environment, both assumptions may not always hold true.

The operator of the UAV-mounted MEC server should enjoy some form of profit by offering its computing services and capabilities to the end-users. Depending on the operation mode, business model and use case under consideration, the profit could be—either explicit or implicit—expressed in different forms (e.g., monetary cost, etc.) [16,17]. In our work, we consider that the profit for the UAV-Mounted MEC server originates directly from applying a usage-based charging policy for allowing the exploitation of the server’s computing resources.

Moreover, it has been argued recently that in real life the end-users are characterized by loss averse and risk seeking behavior in terms of exploiting the system’s available resources, especially in resource-constrained environments (e.g., [18,19,20]). In our case, the resources that the users may opt for and compete for refer to the UAV-mounted MEC server’s computing resources. Specifically, based on the users’ behavioral characteristics, some users may act aggressively and opportunistically in terms of offloading their data to the UAV-mounted MEC servers in order to avoid consuming their personal resources to process their data. Those users exhibit risk seeking behavior, as the UAV-mounted MEC server may not be able to serve all the users’ data offloading and processing requests. On the other hand, there are more conservative users, who exhibit loss averse behavior, thus being more willing to process their data locally at their devices, instead of taking the risk of finally not being served by the UAV-mounted MEC server due to the potential overexploitation of the latter.

Towards jointly addressing the aforementioned assumptions and filling the respective research gaps, in this paper, we exploit the power and principles of Prospect Theory [18] to capture the users risk-based behavior in their data offloading decision-making process, under the operation framework of a usage-based pricing mechanism of the UAV-mounted MEC servers’ computing resources. To the best of our knowledge, this is the first research work in the existing literature that jointly combines a pricing-aware and risk-aware framework to deal with the data offloading problem in UAV-mounted multi-access edge computing systems.

In particular, we assume that the users have two available options for executing their tasks, namely the local computation and the remote computation, the latter achieved through data offloading. The local computation resources of the user’s device act as safe resources, since the users do not compete with each other for consuming those resources. On the other hand, the computation resources of the UAV-mounted MEC server are treated as a Common Pool of Resources (CPR), as they are non-excludable, i.e., all the users have the right to exploit them, while they are rivalrous and subtractable, i.e., their exploitation by one user reduces the ability to be exploited by another user. In principle, the UAV-mounted MEC server resources have the potential to provide significantly higher satisfaction to the user (compared to the lower satisfaction that could be obtained through the limited user local computation resources), if properly utilized and allocated. However, if the users selfishly offload their data to the UAV-mounted MEC server, then the computing capabilities of the latter will be overexploited resulting in suboptimal outcomes for the entire set of users, possibly leading to the complete “failure” of the CPR UAV-mounted MEC server. The failure of the CPR UAV-mounted MEC server refers to its inability to concurrently handle the large amount of offloaded data and corresponding computation tasks by the users, due to its limited computation capability.

To treat this situation and differentiate the performance and usage of the available computation resources, we capitalize on the theory of Tragedy of the Commons [21,22], while also introducing a usage-based pricing mechanism to capture the users’ cost for exploiting the server’s computation resources. The proposed pricing mechanism implicitly introduces a more social behavior to the users and supports the fairness among them, in terms of competing for the UAV-mounted MEC server’s computation resources. The users’ behavioral characteristics in the data offloading decision-making process, is captured through the principles of Prospect Theory that models users’ decisions under the uncertainty of the available computation resources at the UAV-mounted MEC server. Prospect Theory is a well known behavioral economic theory studying the autonomous decision-making of the individuals under risk and uncertainty of the associated payoff of their choices, which is estimated with some probability [23]. The performance evaluation and validation of the proposed pricing and risk-aware data offloading framework in UAV-assisted MEC systems is achieved via modeling and simulation, in terms of efficient exploitation of all the available computation resources, realistic capturing of users’ interaction with the computing environment, and its scalability.

### 1.3. Outline

The rest of this paper is organized as follows. Section 2 presents the considered system model, while Section 3 initially discusses the main principles of Prospect Theory and the theory of the Tragedy of the Commons, and then accordingly designs and formulates the users’ prospect-theoretic utility function. Section 4 introduces the pricing and risk-aware data offloading framework in a UAV-assisted multi-access edge computing system by formulating and solving the corresponding optimization data offloading problem, via adopting the theory of S-modular games. In Section 5, a low complexity and iterative algorithm is introduced to determine the Pure Nash Equilibrium (PNE) of the users’ data offloading optimization problem. Section 6 contains the performance evaluation of the proposed framework, while Section 7 concludes the paper.

## 2. System Model

**Figure 1:**
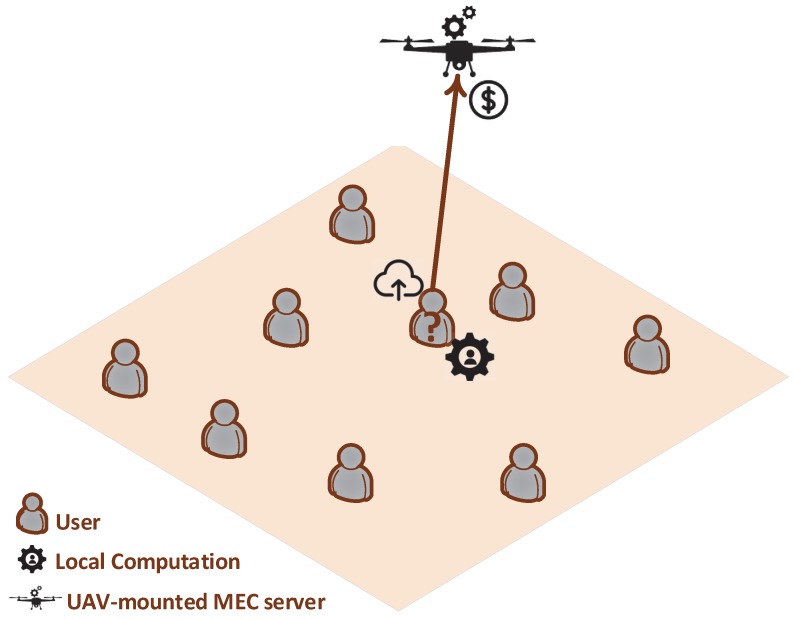
UAV-assisted multi-access edge computing system.

A UAV-assisted multi-access edge computing system is considered consisting of a set of mobile users N={1,…,n,…,N} and a UAV-mounted MEC server attached to the UAV. Each user *n* has a computation task $J_{n}$ that needs to execute. Each task is accordingly defined as $J_{n}=\left(b_{n},d_{n}\right)$, where $b_{n}$ [bits] is the user’s *n* size of the input data needed for the computation task and $d_{n}$ [CPU-cycles] is the number of CPU cycles required in order to accomplish the computation task. The UAV-mounted MEC server is available to the users to offload and process their data remotely instead of processing them locally on their device and consuming their own local resources. Each user decides to offload $b_{n}^{MEC}$ [bits] data to the UAV-mounted MEC server, while the rest ($b_{n}-b_{n}^{MEC}$) [bits] data are processed locally on the user’s device. An indicative topology of the considered UAV-assisted MEC system is presented in Figure 1. In this work we mainly focus on the modeling and provisioning of the computing resources, rather than on the user to UAV wireless communication aspects. The UAV flexibility and adaptability capabilities can ensure strong communication channels and links with the users.

For each user *n*, the time ${\hat{t}}_{n}$ [s] to process the whole amount of data $b_{n}$ locally is defined as:

(1) $${\hat{t}}_{n}=\frac{d_{n}}{f_{n}}$$

where $f_{n}$ [CPU-cycles/s] is the computation capability of each user’s *n* device. Apart from the processing time needed, each computation task has some energy requirements as well. The energy ${\hat{e}}_{n}$ [J] needed to process the whole amount of data $b_{n}$ locally for each user *n* is defined as:

(2) $${\hat{e}}_{n}=\gamma_{n}d_{n}$$

where $\gamma_{n}$ [J/CPU-cycle] is the coefficient denoting the consumed energy per CPU cycle locally at each user’s *n* device.

We assume that the UAV-mounted MEC server applies a fair usage-based pricing policy to the users, while charging them proportionally to their offloaded data and to their demand of consuming computation resources, as they are indicated by the nature of their computation task. Thus, the cost imposed by the UAV-mounted MEC server to the user *n* in order to process the user’s offloaded data $b_{n}^{MEC}$ is defined as:

(3) $$c_{n}\left(b_{n}^{MEC}\right)=cd_{n}\frac{b_{n}^{MEC}}{b_{n}}$$

where *c* [1/CPU-cycles] represents a constant pricing factor imposed by the UAV-mounted MEC server to every user. Intuitively, the cost imposed to each user (Equation (3)) is proportional to the percentage of the number of CPU cycles $d_{n}$ of the user’s computation task that is actually offloaded, i.e., the greater the part of the computation task offloaded to the UAV-mounted MEC server is, the greater is the cost that the user experiences by the UAV-mounted MEC server to process remotely its data. It is noted that, without loss of generality, the cost $c_{n}\left(b_{n}^{MEC}\right)$ imposed by the UAV-mounted MEC server to the user *n* in order to process the offloaded data of the latter is assumed to be a unitless metric in this research work, and can represent any type of usage-based cost or monetary cost in a realistic implementation. Based on the above proposed model, we can therefore formulate the problem of determining the optimal $b_{n}^{MEC*}$ that each user should offload considering each user’s risk-aware behavioral characteristics and the pricing imposed by the UAV-mounted MEC server.

## 3. Users Prospect-Theoretic Utility Function in UAV-Assisted MEC Environment

In the dynamic computation environment considered in this research work, consisting of the UAV-mounted MEC server’s and the users’ local computing capabilities, the users exhibit a risk-aware behavior in terms of deciding where to process the data of their computation tasks. Therefore, the users do not act as risk-neutral utility maximizers following the conventional Expected Utility Theory (EUT) [23], but instead they rather exhibit a loss averse or gain seeking behavior when utilizing the UAV-mounted MEC server’s computation resources. To capture the exploitation and usage characteristics and principles of the available computation resources in the considered UAV-assisted MEC system, we adopt the theory of the Tragedy of the Commons [22]. Specifically, the UAV-mounted MEC server’s computation resources are considered as a Common Pool of Resources (CPR), as all the users have access to them and can offload their data to the UAV-mounted MEC server in order to be processed. If the users overexploit the computation resources of the UAV-mounted MEC server, the latter will fail to serve their computation demands and none of the users will be satisfied. On the other hand, the user’s device’s local computation resources are considered as safe resources, as each user exclusively exploits them for its own benefit. It is noted that the safe resources provide a guaranteed satisfaction to the user; however, the user can potentially experience lower satisfaction compared to exploiting the CPR, as the user has to spend its own resources, e.g., energy to process locally its data.

As mentioned before, towards capturing the users’ loss averse and gain seeking behavior in terms of exploiting the CPR and safe computation resources, the principles of Prospect Theory are adopted [24]. Prospect Theory is a behavioral economic theory that quantifies individuals’ behavioral patterns, which demonstrate systematic deviations from the Expected Utility Theory. Under the prospect theoretic model, the users experience greater dissatisfaction from a potential outcome of losses compared to their satisfaction from gains of the same amount. In addition, the level of the users’ satisfaction and dissatisfaction is evaluated with respect to a reference point, which is considered as the ground truth of the examined system. Recently, several efforts have appeared in the literature, where Prospect Theory has been adopted in various environments and application domains, including dynamic resource management in 5G wireless networks [18,25], public safety networks [19], anti-jamming communications in cognitive radio networks [20], users’ transmission power management and anti-jamming techniques in UAV-assisted networks [5], and Quality of Experience in cyber-physical social systems [21].

Following the principles of Prospect Theory, the user’s prospect theoretic utility is defined as [24,26]:

(4) $$P_{n}\left(U_{n}\right)=\left\{\begin{array}{cc} {\left(U_{n}-U_{n,0}\right)}^{\alpha_{n}}, & \;\mathrm{if}\;U_{n}\geq U_{n,0}\;\\ -k_{n}{\left(U_{n,0}-U_{n}\right)}^{\beta_{n}}, & \;\mathrm{otherwise}\; \end{array}\right.$$

where $U_{n,0}=\frac{1}{{\hat{t}}_{n}{\hat{e}}_{n}}b_{n}$ denotes the reference point expressing the user’s *n* perceived satisfaction by processing all of its data locally at its device, which is the safe choice in terms of receiving a guaranteed satisfaction. Similarly, $U_{n}$ denotes the user’s actual perceived satisfaction from offloading part of its data to the UAV-mounted MEC server, and is given by Equation (5) below.

The parameters $\alpha_{n},\beta_{n}$ where $\alpha_{n},\beta_{n}\in \left(0,1\right]$ express the sensitivity of users to the gains and losses of their actual perceived satisfaction $U_{n}$, respectively. In particular, the user’s risk averse behavior in gains and risk seeking behavior in losses is captured by small values of the parameter $\alpha_{n}\in \left(0,1\right]$. Similarly, a small value of the parameter $\beta_{n}\in \left(0,1\right]$ captures a higher decrease in the user’s prospect theoretic utility, when its actual perceived satisfaction is close to the reference point. It is noted that the values of the parameters $\alpha_{n},\beta_{n}$ can be determined and quantified based on statistical analysis of existing open datasets stemming from qualitative results of users’ behavioral models (e.g., [27]). Furthermore, the loss aversion parameter $k_{n}\in R^{+}$ quantifies the impact of losses compared to the gains in user’s prospect theoretic utility. Specifically, for $k_{n}>1$, the user weighs the losses more than the gains, while the exact opposite holds true for $0\leq k_{n}\leq 1$. For simplicity and without loss of generality, in this work, we assume $\alpha_{n}=\beta_{n}$.

Specifically, the user’s actual perceived satisfaction from offloading part of its data (denoted by $b_{n}^{MEC}$) to the UAV-mounted MEC server is denoted as $U_{n}\left(b_{n}^{MEC}\right)$ and is formally defined as follows:

(5) $$U_{n}\left(b^{\mathrm{MEC}}\right)=\left\{\begin{array}{cc} \frac{1}{{\hat{t}}_{n}{\hat{e}}_{n}}b_{n}, & \;\mathrm{if}\;b_{n}^{MEC}=0\\ \frac{1}{{\hat{t}}_{n}{\hat{e}}_{n}}\left(b_{n}-b_{n}^{MEC}\right)+b_{n}^{MEC}RoR\left(d_{\tau }\right)-c_{n}\left(b_{n}^{MEC}\right), & \;\mathrm{if}\;b_{n}^{MEC}\neq 0\;\mathrm{and}\;\mathrm{MEC}\;\mathrm{survives}\\ \frac{1}{{\hat{t}}_{n}{\hat{e}}_{n}}\left(b_{n}-b_{n}^{MEC}\right)-c_{n}\left(b_{n}^{MEC}\right), & \;\mathrm{if}\;b_{n}^{\mathrm{MEC}}\neq 0\;\mathrm{and}\;\mathrm{MEC}\;\mathrm{fails} \end{array}\right.$$

The first branch of Equation (5) expresses the user’s actual perceived satisfaction from processing all of its data locally to its mobile device. The second branch of Equation (5) captures the user’s actual perceived satisfaction by processing part of its data locally (first term) and part of them to the UAV-mounted MEC server (second term), while experiencing the corresponding usage-based cost (third term) for exploiting the UAV-mounted MEC server’s computation resources in the case that the MEC server can process all the users’ requests. The third branch of Equation (5) represents the user’s utility in the case that the MEC server fails to process the users’ data due to its overexploitation. The user’s actual perceived satisfaction from processing part of its data to the UAV-mounted MEC server depends on the server’s rate of return function $RoR(d_{\tau })$, where $d_{\tau }\left(b^{\mathrm{MEC}}\right)$, $b^{\mathrm{MEC}}=\left(b_{1}^{MEC},\ldots ,b_{N}^{MEC}\right)$ is a normalized increasing function with respect to the users’ total demand of computation resources by the UAV-mounted MEC server. The vector $b^{\mathrm{MEC}}=\left(b_{1}^{MEC},\ldots ,b_{N}^{MEC}\right)$ denotes the data offloading strategies of all the users in the examined system to the UAV-mounted MEC server. For demonstration purposes and without loss of generality, the users’ total demand function $d_{\tau }\left(b^{\mathrm{MEC}}\right)\in \left[0,1\right]$ of computation resources by the UAV-mounted MEC server is defined as follows:

(6) $$d_{\tau }\left(b^{\mathrm{MEC}}\right)=-1+\frac{2}{1+e^{-\theta \sum_{n=1}^{N}d_{n}\frac{b_{n}^{MEC}}{b_{n}}}}$$

where θ>0 is a positive constant calibrating the sigmoidal curve of Equation (6) based on the computing capabilities of the UAV-mounted MEC server. The users’ total computation demand function $d_{\tau }\left(b^{\mathrm{MEC}}\right)$ is a continuous and strictly increasing function with respect to the users’ total amount of offloaded data. Equation (6) is a representative example of the users’ total computation demand function, while any other function that follows the above described properties can be adopted for the following analysis without loss of generality. In a nutshell, the UAV-mounted MEC server’s rate of return function $RoR(d_{\tau })$ provides positive experience, i.e., $RoR(d_{\tau })>0$, if the server has sufficient computation resources to serve the users’ total computation demand $d_{\tau }\left(b^{\mathrm{MEC}}\right)$. The UAV-mounted MEC server’s rate of return function $RoR(d_{\tau })$ is a continuous, monotonically decreasing, and concave function with respect to the users’ total demand of computation resources, since the server’s computation resources assigned to each user and correspondingly the users’ perceived actual satisfaction decrease for increasing values of the users’ total computation demand [28]. For demonstration purposes, in this paper, we adopt an indicative rate of return function that respects all aforementioned properties and is defined as follows:

(7) $$RoR\left(d_{\tau }\right)=2-e^{d_{\tau }-1}$$

Following the above discussion and focusing on the user’s prospect theoretic utility function, as defined in Equation (4), it is noted that the first branch of Equation (4) expresses the user’s *n* risk-aware satisfaction in the case that the UAV-mounted MEC server survives and can support the users’ total computation demand. In that case, each user targets at the maximization of its gains, while, in the opposite case, i.e., the second branch of Equation (4), the user targets at the minimization of its losses, as the UAV-mounted MEC server has failed due to overexploitation.

If the UAV-mounted MEC-server survives, then the user’s actual utility is determined by the second branch of Equation (5), given that the user offloaded part of its data to the MEC server. Thus, in combination with the first branch of Equation (4), the user’s prospect theoretic utility is given as follows:

(8) $$\begin{array}{cc} P_{n}^{surv.}\left(U_{n}\right) & ={\left(U_{n}-U_{n,0}\right)}^{\alpha_{n}}\\ \; & ={\left(b_{n}^{MEC}\right)}^{\alpha_{n}}{\left[\left(2-e^{d_{\tau }-1}\right)-\frac{1}{{\hat{t}}_{n}{\hat{e}}_{n}}-c\frac{d_{n}}{b_{n}}\right]}^{\alpha_{n}} \end{array}$$

If the opposite holds true, that is, the UAV-mounted MEC server’s computation resources are overexploited by the users and the server fails to serve them, then by combining the second branch of Equation (4) and the third branch of Equation (5), the user’s prospect theoretic utility can be written as follows:

(9) $$\begin{array}{cc} P_{n}^{fail}\left(U_{n}\right) & =-k_{n}{\left(U_{n,0}-U_{n}\right)}^{\alpha_{n}}\\ \; & =-k_{n}{\left(b_{n}^{MEC}\right)}^{\alpha_{n}}{\left(\frac{1}{{\hat{t}}_{n}{\hat{e}}_{n}}+c\frac{d_{n}}{b_{n}}\right)}^{\alpha_{n}} \end{array}$$

Furthermore, the probability of failure of the UAV-mounted MEC server, which is the server’s probability to fail serving the users’ total computation demand $d_{\tau }$ (Equation (6)), is denoted by $Pr(d_{\tau })$. The UAV-mounted MEC server’s probability of failure function $Pr\left(d_{\tau }\right),0\leq Pr\left(d_{\tau }\right)\leq 1$ is assumed to be continuous, strictly increasing, convex, and twice differentiable function with respect to the users’ total computation demand $d_{\tau }$. In the following, we adopt the square function to present the UAV-mounted MEC server’s probability of failure, as shown below:

(10) $$Pr\left(d_{\tau }\right)=d_{\tau }^{2}$$

**Figure 2:**
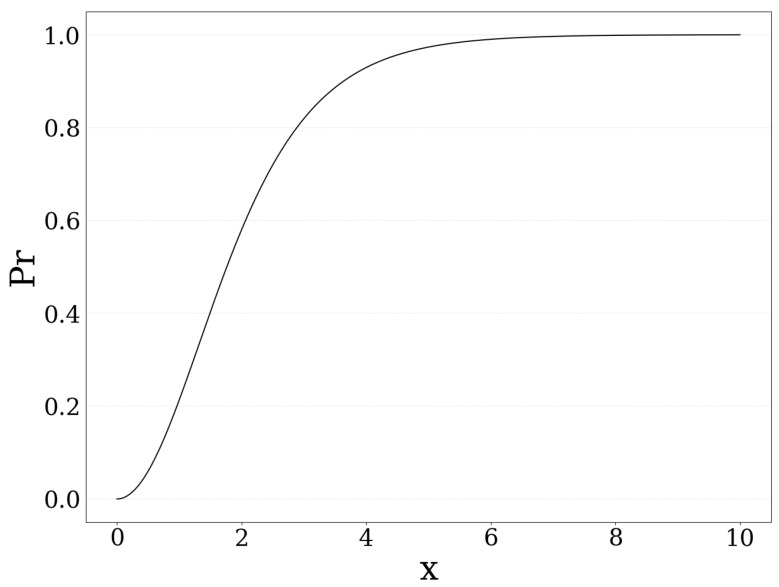
Probability of failure vs *x* when $Pr\left(x\right)={\left(-1+\frac{2}{1+e^{-x}}\right)}^{2}$.

It is noted that the rest of the paper’s analysis still holds true for any probability of failure function that is characterized by the properties described above and the selection of the square function for the probability of failure is mainly made for presentation purposes. Accordingly, the UAV-mounted MEC server’s probability to survive and process the users’ total amount of offloaded data are $\left(1-Pr\left(d_{\tau }\right)\right)$. Moreover, due to the nature of the user’s total computation demand (Equation (6)), the UAV-mounted MEC server’s probability of failure (Equation (10)) is convex on low to medium users’ computation demand and concave on high demand, while it asymptotically converges to one, as shown in Figure 2.

Combining Equations (8)–(10), the user’s expected prospect theoretic utility by offloading $b_{n}^{MEC}$ data to the UAV-mounted MEC server is defined as follows, jointly capturing the uncertainty of the UAV-mounted MEC server’s computation resources, the pricing of the UAV-mounted MEC server, as well as the user’s risk-aware characteristics in its data offloading decision:

(11) $$E\left(U_{n}\right)=P_{n}^{surv.}\left(U_{n}\right)\left(1-Pr\left(d_{\tau }\right)\right)+P_{n}^{fail}\left(U_{n}\right)Pr\left(d_{\tau }\right).$$

## 4. Pricing and Risk-Aware Data Offloading in UAV-Assisted MEC Systems

In this section, the distributed pricing and risk-aware data offloading problem in UAV-assisted multi-access edge computing systems is formulated by adopting the principles of non-cooperative game theory and solved based on the theory of S-modular games.

### 4.1. Problem Formulation

Each user aims at maximizing its expected prospect theoretic utility function (Equation (11)) by distribution and autonomously deciding its optimal data offloading strategy $b_{n}^{MEC*}$ to the UAV-mounted MEC server, while considering the imposed pricing policy and its personal risk-aware characteristics. Accordingly, the users’ pricing and risk-aware data offloading problem is formulated as a distributed optimization problem as follows:

(12a) $$\begin{array}{c} \max_{b_{n}^{MEC}\in \left[0,b_{n}\right]}E\left(U_{n}\left(b_{n}^{MEC},b_{-n}^{\mathrm{MEC}}\right)\right) \end{array}$$

(12b) $$\begin{array}{c} s.t.\;\;\;\;0\leq b_{n}^{MEC}\leq b_{n} \end{array}$$

where $b_{-n}^{\mathrm{MEC}}$ denotes the amount of the offloaded data by the rest of the users except for user *n*.

The distributed optimization problem of users’ data offloading can be formulated as a non-cooperative game among the users $G=[N,A_{n},E\left(U_{n}\left(b_{n}^{MEC},b_{-n}^{\mathrm{MEC}}\right)\right)]$, where N is the set of users, $A_{n}=\left[0,b_{n}\right]$ is the user’s *n* data offloading strategy space, and $E(U_{n}\left(b_{n}^{MEC},b_{-n}^{\mathrm{MEC}}\right))$ denotes the user’s *n* expected prospect theoretic utility function, as defined in the previous section. The solution of the non-cooperative game *G* should determine each user’s optimal data offloading strategy $b_{n}^{MEC*}$ in order to maximize its expected prospect theoretic utility. The Pure Nash Equilibrium (PNE) approach is adopted and described below, towards analytically seeking the solution of the pricing and risk-aware data offloading problem (Equation (12a) and (12b)).

**Definition 1.**  *(Pure Nash Equilibrium Point): A data offloading vector* $b_{n}^{\mathrm{MEC}*}=\left(b_{1}^{MEC*},\ldots ,b_{N}^{MEC*}\right)$ *in the strategy space* $b_{n}^{MEC*}\in A_{n}=\left[0,b_{n}\right]$ *is a Pure Nash Equilibrium point if for every user n the following condition holds true:*

(13) $$E\left(U_{n}\left(b_{n}^{MEC*},b_{-n}^{\mathrm{MEC}*}\right)\right)\geq E\left(U_{n}\left(b_{n}^{MEC},b_{-n}^{\mathrm{MEC}*}\right)\right)$$

*for all* $b_{n}^{MEC}\in A_{n}$ *.*

The physical interpretation of the above definition is that, at the Pure Nash Equilibrium point, no user has the incentive to unilaterally change its data offloading strategy to the UAV-mounted MEC server given the data offloading strategies of the rest of the users, as its achieved expected prospect theoretic utility cannot be improved.

### 4.2. Problem Solution

In order to prove the existence of at least one PNE of the non-cooperative game *G*, as a solution of the maximization problem (Equation (12a) and (12b)), the theory of submodular games is adopted [29]. The submodular games are characterized by strategic substitutes, i.e., when a user offloads more data to the UAV-mounted MEC server, the rest of the users tend to avoid following similar behavior, as the UAV-mounted MEC server’s computation resources can become overexploited and none of the users be satisfied. The submodular games are of great interest and practical importance as an optimization tool, due to the fact that they guarantee the existence of at least one PNE, while learning and adjustment tools (such as the best response dynamics) can be used in order to determine such a point.

**Definition 2.**  *(Submodular Games): The non-cooperative game* $G=[N,A_{n},E\left(U_{n}\left(b_{n}^{MEC},b_{-n}^{\mathrm{MEC}}\right)\right)]$ *is submodular, if, for all the users, the following conditions hold true [30]:*

*1.*  $A_{n}$ *is a compact subset of an Euclidean space.*
*2.*  $E(U_{n}\left(b_{n}^{MEC},b_{-n}^{\mathrm{MEC}}\right))$ *is smooth, submodular in* $b_{n}^{MEC}$ *, and has non-increasing differences in* $\left(b_{n}^{MEC},b_{-n}^{\mathrm{MEC}}\right)$ *, i.e.,* $\frac{\partial^{2}E_{n}\left({\vec{b}}^{MEC}\right)}{\partial b_{j}^{MEC}\partial b_{n}^{MEC}}\leq 0$ *.*

Additionally, in a submodular game, there always exist external equilibria [31]: a largest best response strategy $\bar{b_{n}^{MEC}}=sup\left\{b_{n}^{MEC}\in A_{n}:BR\left(b_{n}^{MEC},b_{-n}^{\mathrm{MEC}}\right)\geq b_{n}^{MEC}\right\}$ and a smallest best response strategy: $\underline{b_{n}^{MEC}}=inf\left\{b_{n}^{MEC}\in A_{n}:BR\left(b_{n}^{MEC},b_{-n}^{\mathrm{MEC}}\right)\leq b_{n}^{MEC}\right\}$ of the non-empty set of Pure Nash Equilibria, where $BR(b_{n}^{MEC},b_{-n}^{\mathrm{MEC}})$ denotes the user’s *n* best response strategy to the other users’ strategies.

**Theorem** **1.** *The non-cooperative game* $G=[N,A_{n},E\left(U_{n}\left(b_{n}^{MEC},b_{-n}^{\mathrm{MEC}}\right)\right)]$ *is submodular for all* $d_{\tau }\in \left(0,\mu \right)$ *, where* μ∈(0,1) *, and* $c<\frac{b_{n}}{d_{n}}\left(1-\frac{1}{{\hat{t}}_{n}{\hat{e}}_{n}}\right)$ *, and has at least one Pure Nash Equilibrium point.*

**Proof.**  The strategy space $A_{n}=\left[0,b_{n}\right]$ is a compact subset of a Euclidean space. The user’s expected prospect theoretic utility function $E(U_{n}\left(b_{n}^{MEC},b_{-n}^{\mathrm{MEC}}\right))$, as defined in Equation (11), is smooth, as it has derivatives of all orders everywhere in its domain $A_{n}$. Towards showing that the user’s expected prospect theoretic utility function is submodular in $b_{n}$ and has non-increasing differences in $\left(b_{n}^{MEC},b_{-n}^{\mathrm{MEC}}\right)$, we examine the properties of the second order partial derivative of the user’s expected prospect theoretic utility function, i.e., $\frac{\partial^{2}E_{n}\left({\vec{b}}^{MEC}\right)}{\partial b_{j}^{MEC}\partial b_{n}^{MEC}}\leq 0$.

We can rewrite Equation (11) using Equations (8) and (9), as follows:

(14) $$E\left(U_{n}\left(b_{n}^{MEC},b_{-n}^{\mathrm{MEC}}\right)\right)={\left(b_{n}^{MEC}\right)}^{\alpha_{n}}\left\{{\left[\left(2-e^{d_{\tau }-1}\right)-\frac{1}{{\hat{t}}_{n}{\hat{e}}_{n}}-c\frac{d_{n}}{b_{n}}\right]}^{\alpha_{n}}\left(1-Pr\left(d_{\tau }\right)\right)-k_{n}{\left(\frac{1}{{\hat{t}}_{n}{\hat{e}}_{n}}+c\frac{d_{n}}{b_{n}}\right)}^{\alpha_{n}}Pr\left(d_{\tau }\right)\right\}$$

We define $\bar{RoR}\left(d_{\tau }\right)={\left[\left(2-e^{d_{\tau }-1}\right)-\frac{1}{{\hat{t}}_{n}{\hat{e}}_{n}}-c\frac{d_{n}}{b_{n}}\right]}^{\alpha_{n}}$ as the user’s specific rate of return, which should be positive in order for the user to have an incentive to offload part of its data to the UAV-mounted MEC server. From Equation (7), the UAV-mounted MEC server’s rate of return function $RoR(d_{\tau })$ is decreasing. Thus, the minimum value of $RoR(d_{\tau })$, and correspondingly of the function $\bar{RoR}\left(d_{\tau }\right)$, is determined at $d_{\tau }=1$. The physical notion of $d_{\tau }=1$ is that all the users offload their total amount of data to the UAV-mounted MEC server for further processing. Following this observation, we can determine the boundaries of the constant pricing factor *c* that the UAV-mounted MEC server imposes on the users, in order for the latter to still have an incentive to offload part of their data to the MEC server without the imposed pricing to become a prohibitive factor. Therefore, the feasible boundaries of the constant pricing factor are determined as follows:

(15) $$\bar{RoR}\left(d_{\tau }=1\right)>0\Rightarrow c<\frac{b_{n}}{d_{n}}\left(1-\frac{1}{{\hat{t}}_{n}{\hat{e}}_{n}}\right)$$

In addition, the following conditions hold true by performing the corresponding derivations: $\frac{\partial d_{\tau }}{\partial b_{n}^{MEC}}>0,\frac{\partial d_{\tau }}{\partial b_{j}^{MEC}}>0,\frac{\partial \bar{RoR}\left(d_{\tau }\right)}{\partial b_{n}^{MEC}}<0,\frac{\partial \bar{RoR}\left(d_{\tau }\right)}{\partial b_{j}^{MEC}}<0,\frac{\partial^{2}\bar{RoR}\left(d_{\tau }\right)}{\partial b_{j}^{MEC}\partial b_{n}^{MEC}}<0,\frac{\partial Pr(d_{\tau })}{\partial b_{n}^{MEC}}>0,\frac{\partial Pr(d_{\tau })}{\partial b_{j}^{MEC}}>0,\frac{\partial^{2}Pr\left(d_{\tau }\right)}{\partial b_{n}^{MEC}\partial b_{j}^{MEC}}=0$. For notational convenience, we set $A=k_{n}{\left(\frac{1}{{\hat{t}}_{n}{\hat{e}}_{n}}+c\frac{d_{n}}{a_{n}}\right)}^{\alpha_{n}}>0$, and we calculate the second order partial derivative of the user’s expected prospect theoretic utility function, as follows:

(16) $$\begin{array}{cc} \frac{\partial^{2}E\left(U_{n}\left(b_{n}^{MEC},b_{-n}^{\mathrm{MEC}}\right)\right)}{\partial b_{j}^{MEC}\partial b_{n}^{MEC}}= & \alpha_{n}{\left(b_{n}^{MEC}\right)}^{\alpha_{n}-1}\left\{\frac{\partial \bar{RoR}\left(d_{\tau }\right)}{\partial b_{j}^{MEC}}\left[1-Pr\left(d_{\tau }\right)\right]-\bar{RoR}\left(d_{\tau }\right)\frac{\partial Pr(d_{\tau })}{\partial b_{j}^{MEC}}-A\frac{\partial Pr(d_{\tau })}{\partial b_{j}^{MEC}}\right\}+\\ \; & {\left(b_{n}^{MEC}\right)}^{\alpha_{n}}\left\{\frac{\partial^{2}\bar{RoR}\left(d_{\tau }\right)}{\partial b_{j}^{MEC}\partial b_{n}^{MEC}}\left[1-Pr\left(d_{\tau }\right)\right]-\frac{\partial \bar{RoR}\left(d_{\tau }\right)}{\partial b_{n}^{MEC}}\frac{\partial Pr(d_{\tau })}{\partial b_{j}^{MEC}}-\frac{\partial \bar{RoR}\left(d_{\tau }\right)}{\partial b_{j}^{MEC}}\frac{\partial Pr(d_{\tau })}{\partial b_{n}^{MEC}}\right\}\\ = & {\left(b_{n}^{MEC}\right)}^{\alpha_{n}-1}\{\alpha_{n}\frac{\partial \bar{RoR}\left(d_{\tau }\right)}{\partial b_{j}^{MEC}}\left[1-Pr\left(d_{\tau }\right)\right]-\alpha_{n}\bar{RoR}\left(d_{\tau }\right)\frac{\partial Pr(d_{\tau })}{\partial b_{j}^{MEC}}-A\alpha_{n}\frac{\partial Pr(d_{\tau })}{\partial b_{j}^{MEC}}+\\ \; & b_{n}^{MEC}\frac{\partial^{2}\bar{RoR}\left(d_{\tau }\right)}{\partial b_{j}^{MEC}\partial b_{n}^{MEC}}\left[1-Pr\left(d_{\tau }\right)\right]-b_{n}^{MEC}\frac{\partial \bar{RoR}\left(d_{\tau }\right)}{\partial b_{n}^{MEC}}\frac{\partial Pr(d_{\tau })}{\partial b_{j}^{MEC}}-b_{n}^{MEC}\frac{\partial \bar{RoR}\left(d_{\tau }\right)}{\partial b_{j}^{MEC}}\frac{\partial Pr(d_{\tau })}{\partial b_{n}^{MEC}}\} \end{array}$$

Let $\psi \left(d_{\tau }\right)=\frac{\partial \bar{RoR}\left(d_{\tau }\right)}{\partial b_{j}^{MEC}}\left[\alpha_{n}-\alpha_{n}Pr\left(d_{\tau }\right)-b_{n}^{MEC}\frac{\partial Pr(d_{\tau })}{\partial b_{n}^{MEC}}\right]-b_{n}^{MEC}\frac{\partial \bar{RoR}\left(d_{\tau }\right)}{\partial b_{n}^{MEC}}\frac{\partial Pr(d_{\tau })}{\partial b_{j}^{MEC}}$. We can rewrite Equation (16), as follows:

(17) $$\frac{\partial^{2}E\left(U_{n}\left(b_{n}^{MEC},b_{-n}^{\mathrm{MEC}}\right)\right)}{\partial b_{j}^{MEC}\partial b_{n}^{MEC}}={\left(b_{n}^{MEC}\right)}^{\alpha_{n}-1}\left\{\psi \left(d_{\tau }\right)-\alpha_{n}\bar{RoR}\left(d_{\tau }\right)\frac{\partial Pr(d_{\tau })}{\partial b_{j}^{MEC}}-A\alpha_{n}\frac{\partial Pr(d_{\tau })}{\partial b_{j}^{MEC}}+b_{n}^{MEC}\frac{\partial^{2}\bar{RoR}\left(d_{\tau }\right)}{\partial b_{j}^{MEC}\partial b_{n}^{MEC}}\left[1-Pr\left(d_{\tau }\right)\right]\right\}$$

It is observed that the last three terms of Equation (17) are negative; thus, we study the properties of the function $\psi (d_{\tau }),\forall n\in N$. For $d_{\tau }=0$, we have $b_{n}^{MEC}=0$. Thus, we calculate:

(18) $$\psi \left(d_{\tau }=0\right)=\frac{\partial \bar{RoR}\left(0\right)}{\partial b_{j}^{MEC}}\alpha_{n}<0$$

For $d_{\tau }\approx 1$, we have $b_{n}^{MEC}=b_{n},\forall n\in N$. Thus, we calculate:

(19) $$\psi \left(d_{\tau }\approx 1\right)-b_{n}\left[\frac{\partial \bar{RoR}\left(1\right)}{\partial b_{j}^{MEC}}\frac{\partial Pr(1)}{\partial b_{n}^{MEC}}+\frac{\partial \bar{RoR}\left(1\right)}{\partial b_{n}^{MEC}}\frac{\partial Pr(1)}{\partial b_{j}^{MEC}}\right]>0$$

Since $\psi (d_{\tau })$ is continuous, using the Bolzano Theorem [32], we conclude that there exists at least one μ∈(0,1) such that $\psi (d_{\tau }=\mu )=0$. Given that $\psi (d_{\tau }=0)<0$ (Equation (18)), then, if μ is the smallest possible value in (0,1) such that $\psi (d_{\tau }=\mu )=0$, then $\psi \left(d_{\tau }\right)<0,\forall d_{\tau }\in \left(0,\mu \right)$. Thus, we conclude that

(20) $$\frac{\partial^{2}E\left(U_{n}\left(b_{n}^{MEC},b_{-n}^{\mathrm{MEC}}\right)\right)}{\partial b_{j}^{MEC}\partial b_{n}^{MEC}}<0,\forall d_{\tau }\in \left(0,\mu \right),\mu \in \left(0,1\right).$$

Thus, the non-cooperative game *G* is submodular $\forall d_{\tau }\in \left(0,\mu \right),\mu \in \left(0,1\right)$ and $c<\frac{b_{n}}{d_{n}}\left(1-\frac{1}{{\hat{t}}_{n}{\hat{e}}_{n}}\right)$. Therefore, the non-cooperative game $G=[N,A_{n},E\left(U_{n}\left(b_{n}^{MEC},b_{-n}^{\mathrm{MEC}}\right)\right)]$ has at least one Pure Nash Equilibrium point $b_{n}^{\mathrm{MEC}*}=\left(b_{1}^{MEC*},\ldots ,b_{N}^{MEC*}\right)$ [33]. □

## 5. Pricing and Risk-Aware Distributed Data Offloading Algorithm

Towards enabling the users to determine their optimal data offloading strategy $b_{n}^{MEC*}$ in a distributed manner, the Best Response Dynamics (BRD) approach is adopted. The best response strategy of each user subject to the selected data offloading strategies of the rest of the users is formally determined as follows:

(21) $$BR\left(b_{n}^{MEC},b_{-n}^{\mathrm{MEC}}\right)=b_{n}^{MEC*}=\underset{b_{n}^{MEC}\in \left[0,b_{n}\right]}{arg max}E\left(U_{n}\left(b_{n}^{MEC},b_{-n}^{\mathrm{MEC}}\right)\right).$$

Given that we have already proven that the non-cooperative game $G=[N,A_{n},E\left(U_{n}\left(b_{n}^{MEC},b_{-n}^{\mathrm{MEC}}\right)\right)]$ belongs to the class of submodular games as stated above, and therefore possesses at least one PNE point, it also readily follows that the iterated best-response dynamics always converges to a Pure Nash Equilibrium point [34,35].

Subsequently, capitalizing on the above argumentation, a distributed iterative and low-complexity algorithm is introduced in order to determine the users’ optimal data offloading strategies to the UAV-mounted MEC server (see Algorithm 1). The proposed algorithm follows the philosophy and principles of the best response dynamics learning mechanism, and, at each iteration, each user aims at maximizing its expected prospect theoretic utility given the data offloading strategies of the rest if the users. The complexity of the pricing and risk-aware data offloading algorithm is O(N∗Ite∗A), where Ite is the total number of iterations in order for the algorithm to converge to the PNE, and *A* is the complexity of solving Equation (21). Detailed numerical results regarding the operation performance and scalability of our approach and algorithm, in terms of iterations, are presented in the following section as well.

**Algorithm 1** Pricing and risk-aware data offloading algorithm
**Input:***N*, *c*, $b_{n}$, $d_{n}$, $f_{n}$, $\gamma_{n}$, ∀n∈N
**Output:** $b^{\mathrm{MEC}*}$
**Initialization: **ite=0, Convergence=**false**, $b_{n}^{{MEC}^{\left(ite=0\right)}}$, ∀n∈N
**while** Convergence== **false do**
 ite=ite+1; **for **n=1 to *N* **do**  user *n* determines $b_{n}^{{MEC*}^{\left(ite\right)}}$ w.r.t. $b_{n}^{{MEC*}^{\left(ite-1\right)}}$ (Equation (21)) and receives $E{\left(U_{n}\right)}^{\left(ite\right)}$ **end for** **if **$b_{n}^{{MEC*}^{\left(ite\right)}}$=$b_{n}^{{MEC*}^{\left(ite-1\right)}}$**then**  
Convergence=**true**
 **end if**
**end while**



## 6. Numerical Results

In this section, we provide a series of numerical results, obtained via modeling and simulation, evaluating the performance and the inherent attributes of the proposed pricing and risk-aware data offloading framework. Initially, the pure operational characteristics of the proposed framework are presented (Section 6.1), while the impact of the introduced usage-based pricing scheme is quantified and studied (Section 6.2). Moreover, a scalability analysis of the proposed framework is performed in Section 6.3, while the impact of the prospect theoretic parameters reflecting the user behavioral pattern in terms of loss aversion and sensitivity, on the overall system performance is evaluated in Section 6.4. The performed simulations were executed on an Intel Core i5-4300U CPU @ 1.90 GHz × 4 with 8 GB RAM (New York, NY, USA). The main parameters used in our simulation, along with their typical values, are presented in Table 1. In the rest of the analysis, and in particular in Section 6.1 and Section 6.2, we have considered N=25 users, and sensitivity ($k_{n}$) and loss aversion ($\alpha_{n}$) parameter values as indicated in Table 1. However, in Section 6.3 and Section 6.4, a wider range of the number of users and the loss aversion and sensitivity parameters are considered.

### 6.1. Pure Operation of the Framework

**Figure 3:**
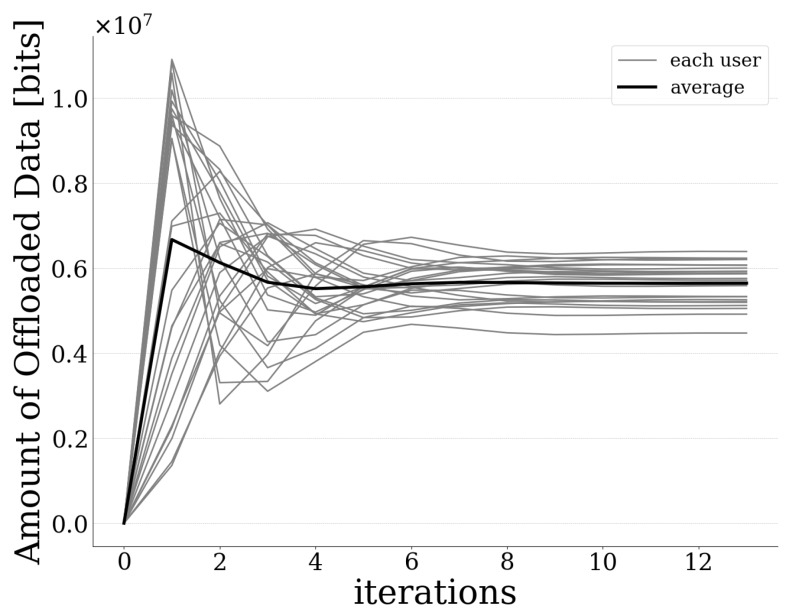
Amount of data offloaded by each user vs. iterations.

**Figure 4:**
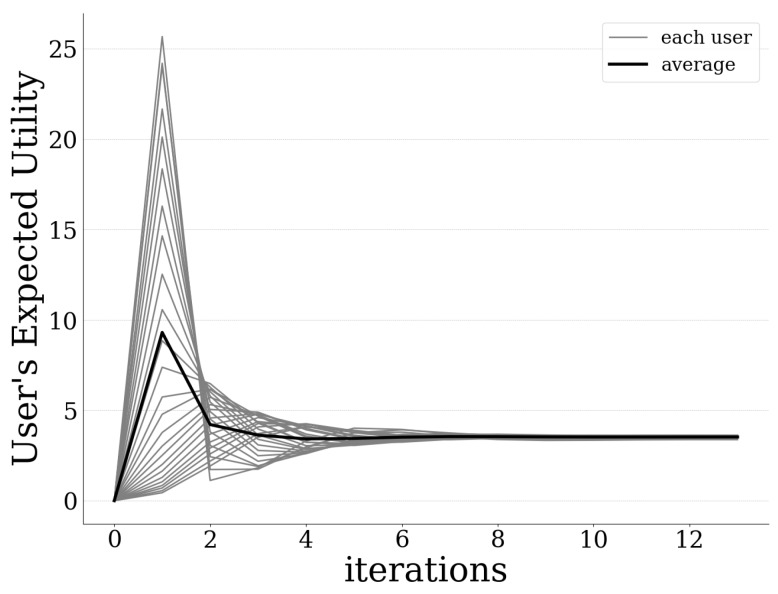
Expected utility of each user vs. iterations.

**Figure 5:**
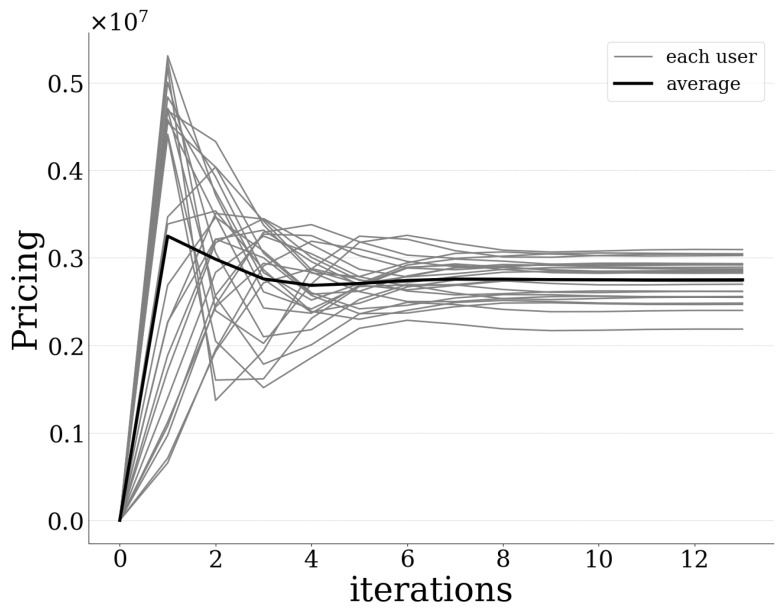
Pricing imposed by the server on each user vs. iterations.

Figure 3 presents the amount of offloaded data by each user to the UAV-mounted MEC server, as well as the average amount of offloaded data as a function of the pricing and risk-aware data offloading algorithm’s iterations. The results reveal that the introduced best response dynamics-based algorithm converges to the PNE quite fast and in small iterations (less than 10 iterations are required for all users). Moreover, Figure 4 and Figure 5 illustrate each user’s expected prospect-theoretic utility and the corresponding usage-based pricing imposed by the UAV-mounted MEC server as a function of the algorithm’s iterations. The corresponding results reveal that initially the users tend to offload a great portion of their data to the MEC server, as observed in Figure 3, and therefore their expected prospect-theoretic utility increases (Figure 4). Specifically, at the first iteration of the algorithm, the users present an aggressive behavior in terms of offloading a large amount of data to the UAV-mounted MEC server (Figure 3) towards enjoying a high expected utility. (Figure 4). However, at the same time, this behavior is expected to lead to the increase of the probability of failure of the UAV-mounted MEC server (as it is confirmed below in Figure 7), and accordingly to the users being penalized with a high price. This is demonstrated in Figure 5, where, due to the fact that the users exploit more the computing capabilities of the MEC server, the latter imposes on them a higher usage-based pricing. Consequently, in combination with the impact of probability of failure and rate of return, as the iterations evolve, the users decrease the amount of data that they offload to the MEC server (Figure 3) following the learning mechanism of the best response dynamics, in order to experience a lower pricing (Figure 5) and finally they converge to the PNE.

**Figure 6:**
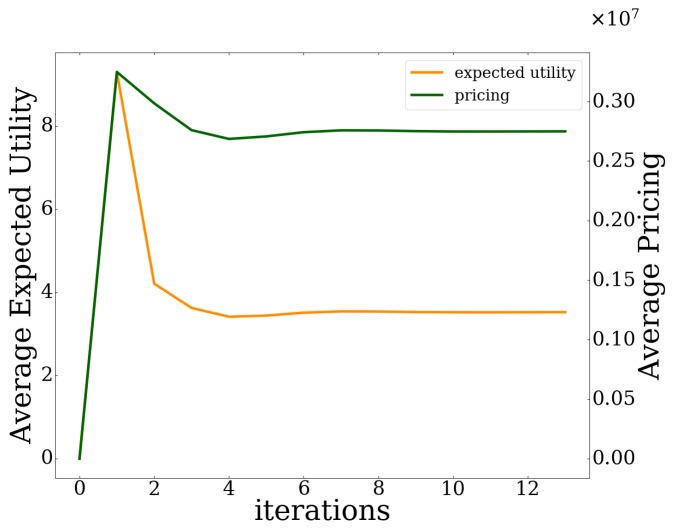
Average users’ expected utility and average users’ pricing vs. iterations.

**Figure 7:**
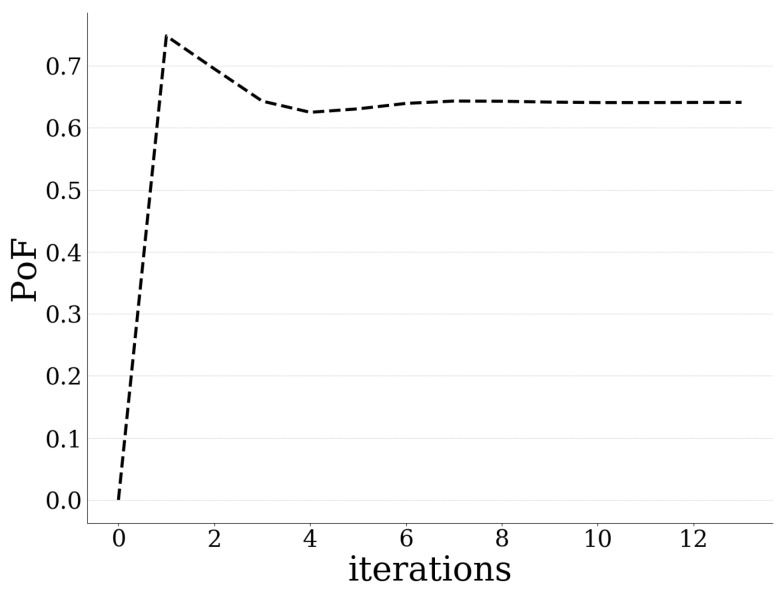
Probability of failure of MEC server vs. iterations.

Figure 6 depicts the users’ average expected prospect-theoretic utility and the users’ average experienced usage-based pricing for exploiting the UAV-mounted MEC server’s computing capabilities, as a function of the algorithm’s iterations. In addition, Figure 7 presents the UAV-mounted MEC server’s probability of failure as a function of the algorithm’s iterations. The above described trend in users’ data offloading strategies is observed from the system’s point of view. Specifically, all the users tend initially to aggressively offload a large amount of data to the MEC server in order to achieve a greater utility (Figure 6). However, the probability of failure of the UAV-mounted MEC server increases due to the over-exploitation of its computing capabilities (Figure 7). Thus, the MEC server imposes a higher pricing on the users (Figure 6) to control their greedy and selfish data offloading behavior.

### 6.2. Impact of Usage-Based Pricing

**Figure 8:**
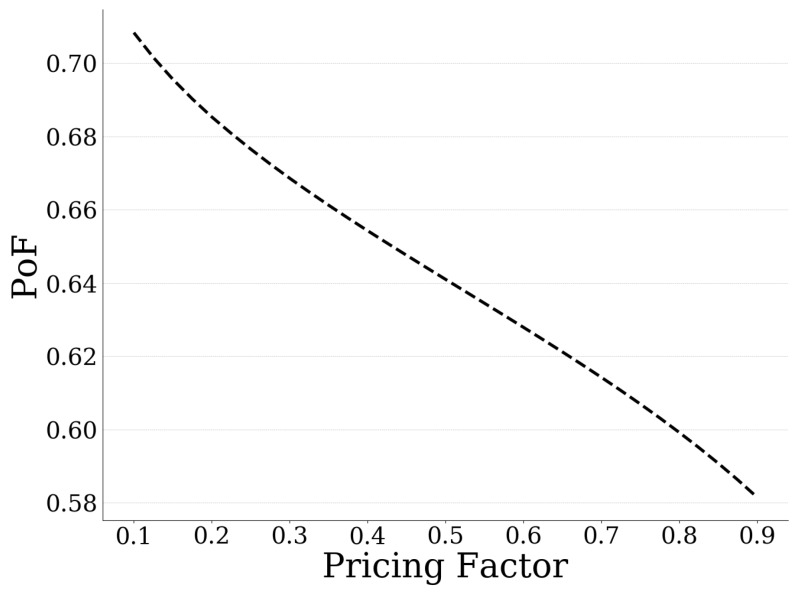
Probability of failure of MEC server vs. the pricing factor.

**Figure 9:**
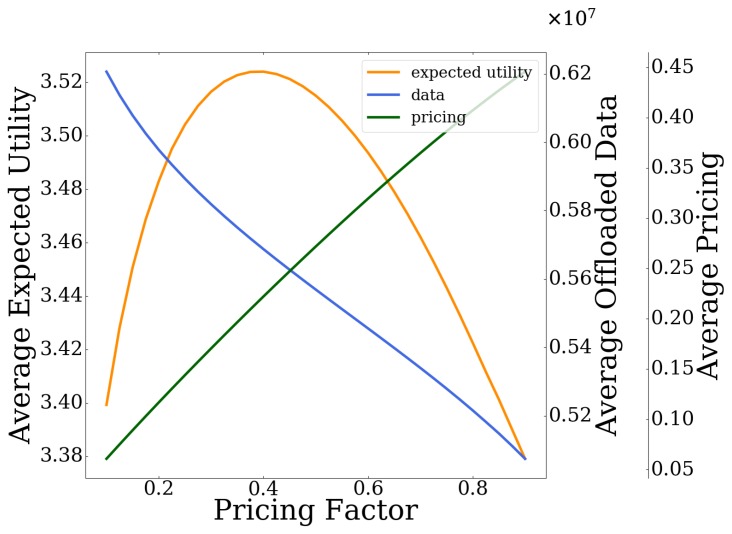
Average expected utility, offloaded data, and pricing vs. the pricing factor.

In this section, we study the impact of the usage-based pricing imposed by the UAV-mounted MEC server, on the users’ data offloading strategies, as well as on the overall operation of the system. Specifically, Figure 8 presents the probability of failure of the MEC server as a function of the pricing factor *c* (Equation (3)). Moreover, the users’ average expected utility, the users’ average amount of offloaded data, and the pricing imposed by the MEC server are presented in Figure 9, as a function of the pricing factor *c* as well. The results reveal that, as the pricing policy becomes stricter (i.e., increasing values of the pricing factor), the usage-based pricing experienced by the users increases (Figure 9) and the exploitation of the MEC server’s computing capabilities becomes cost inefficient after some point (with respect to the total offloaded data). Consequently, the users tend to offload a smaller amount of data to the MEC server (Figure 9), and the MEC server becomes less congested in terms of processing the users’ computation tasks, and its probability of failure decreases (Figure 8).

Based on the results presented in Figure 9, it is observed that the users’ average expected utility is concave with respect to the pricing factor. Specifically, small values of the pricing factor correspond to less-strict pricing policies; thus, the users over-exploit the MEC server’s computing capabilities (i.e., high values of MEC server’s probability of failure are observed), resulting in low values of expected utility. On the other hand, high values of the pricing factor result in discouraging the users to exploit the UAV-mounted MEC server’s computing capabilities, thus concluding again to low levels of users’ average expected utility. Therefore, a balanced pricing policy is required to keep the quality of experience of the users at high levels.

### 6.3. Scalability Evaluation

**Figure 10:**
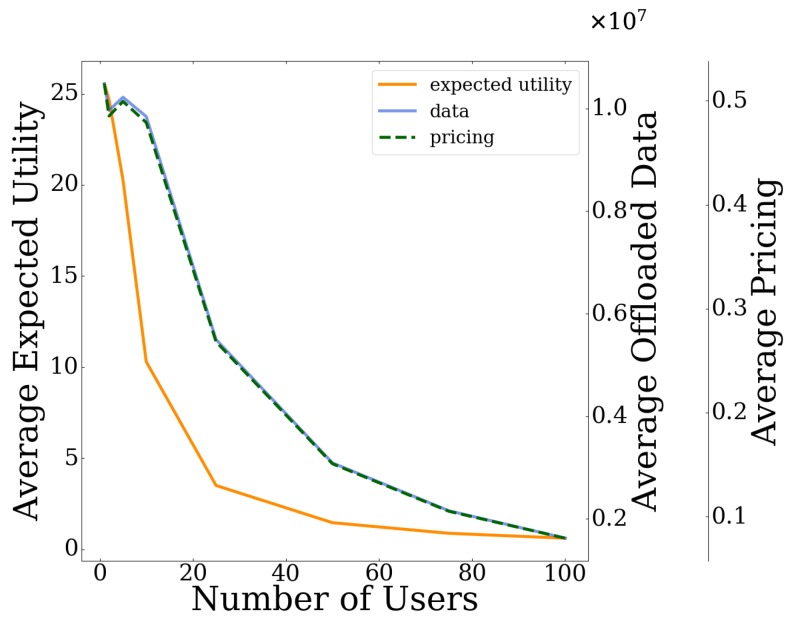
Users’ average expected utility, users’ average offloaded data and pricing at the PNE vs. number of users on the system.

**Figure 11:**
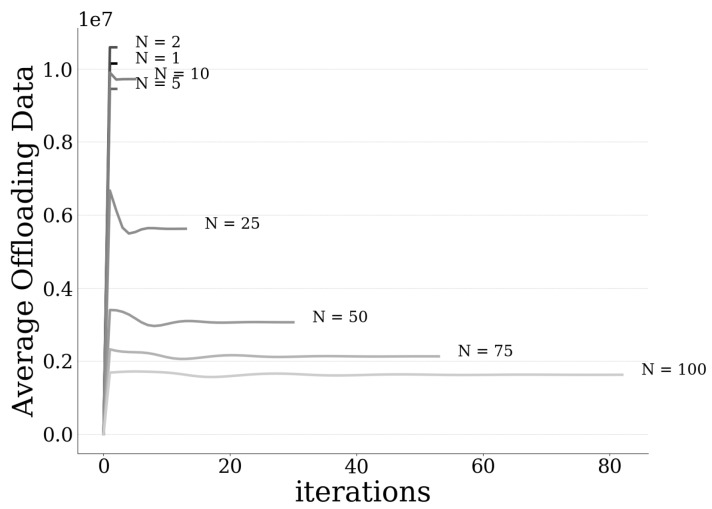
Users’ average data offloading vs. iterations for different numbers of users.

In this section, a scalability evaluation of the proposed pricing and risk-aware data offloading framework is provided considering an increasing number of users in the system. Table 2 presents the iterations and the overall corresponding execution time of the proposed algorithm in order to converge to the PNE point. Given the distributed nature of the best response dynamics approach, we observe that its execution time scales quite well for increasing number of users, achieving a close to real-time implementation in realistic scenarios. Respectively, the users’ average expected utility, the users’ average amount of offloaded data, and the imposed pricing by the UAV-mounted MEC server are presented in Figure 10, as a function of the number of users. The scalability evaluation is complemented by the results presented in Figure 11 that depict the convergence of the users’ average amount of offloaded data as a function of the required number of iterations, for different numbers of users. In particular, we observe that, as the number of users in the system increases, they tend to offload a lower average amount of data to the MEC server (Figure 10 and Figure 11), as the latter becomes over-congested. Thus, they experience both lower pricing (Figure 10) and lower expected utility (Figure 10), as they drive themselves in processing more data locally on their local devices and accordingly consume their own resources, i.e., battery. It is also observed that the user’s experienced pricing $c_{n}\left(b_{n}^{MEC}\right)$ and the user’s offloaded data $b_{n}^{MEC}$ (Figure 10) has the same trend, due to their one-to-one relationship stemming from Equation (3), while the corresponding curves also appear to be overlapping. However, it should be noted here that the actual values for the two curves are different, since there are two different right vertical axes in Figure 10 (each one reflecting the values of each curve respectively).

### 6.4. Impact of Prospect Theoretic Parameters and User Competition

In the following, the impact of the prospect theoretic parameters, reflecting the user behavioral pattern in terms of loss aversion and sensitivity, on the overall system performance is evaluated.

**Figure 12:**
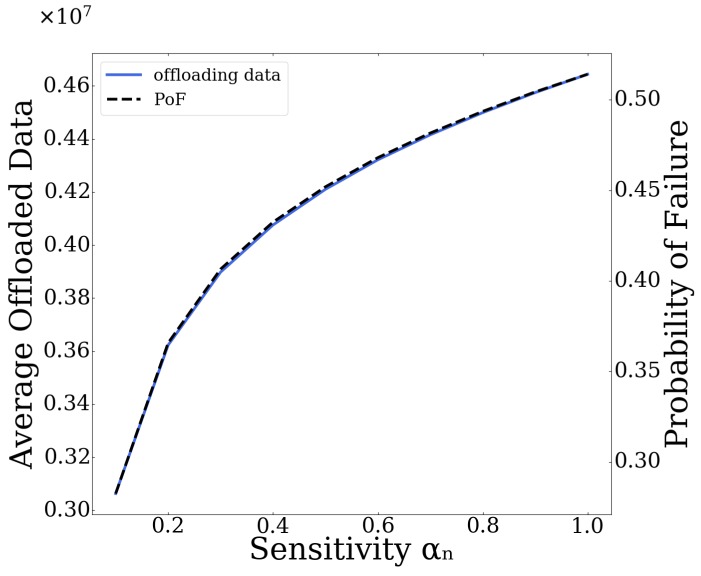
Average offloading data and PoF vs. sensitivity parameter $\alpha_{n}$.

**Figure 13:**
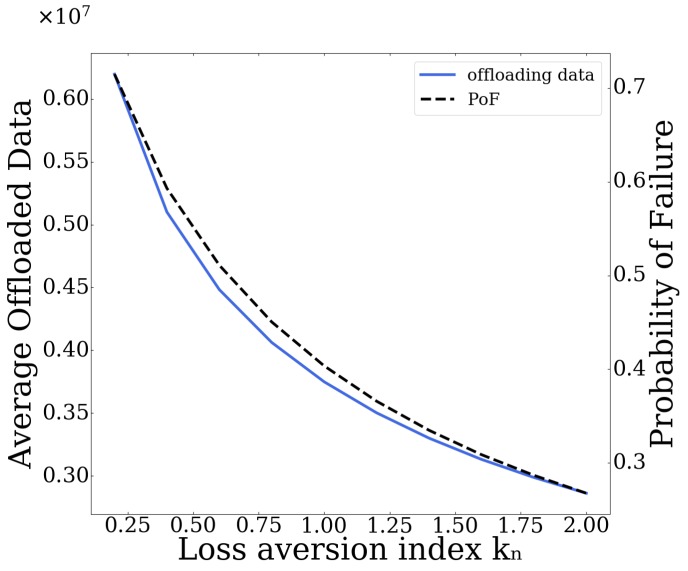
Average offloading data and PoF vs. loss aversion index $k_{n}$.

Specifically, in Figure 12 and Figure 13, initially we present the average user offloaded data and corresponding Probability of failure, as functions of the sensitivity parameter $\alpha_{n}$ and the loss aversion index $k_{n}$, respectively. As can be seen from Figure 12, by increasing the sensitivity parameter $\alpha_{n}$, the users tend to offload more data to the MEC server since they opt to value more the larger gains, compared to those of smaller magnitude. The increased volume of data offloaded results in an increase in the corresponding Probability of Failure of the server as well. In Figure 13, on the other hand, we can see that, as the loss aversion index $k_{n}$ increases, less data are offloaded to the server, since higher value signifies more loss aversion for the users, resulting in smaller Probabilities of Failure of the server.

In order to further study the effect of competition of users for the CPR (i.e., UAV-mounted MEC server), we use the Fragility under Competition (FuC) metric [38]. This metric is expressed as the ratio between the Probability of Failure of the MEC server when *N* users are competing for the MEC server’s resources at the equilibrium state, versus the Probability of Failure of the MEC server when there is only one user offloading data. Formally, the Fragility under Competition is defined as: $FuC=\frac{Pr(b_{N}^{{\mathrm{MEC}}^{*}})}{Pr(b_{1}^{{\mathrm{MEC}}^{*}})}$, where $b_{N}^{MEC^{*}}$ denotes the equilibrium point when *N* users are present and $b_{1}^{MEC^{*}}$ denotes the corresponding equilibrium point if only one user was present, with the same risk preferences as the group of *N* users.

**Figure 14:**
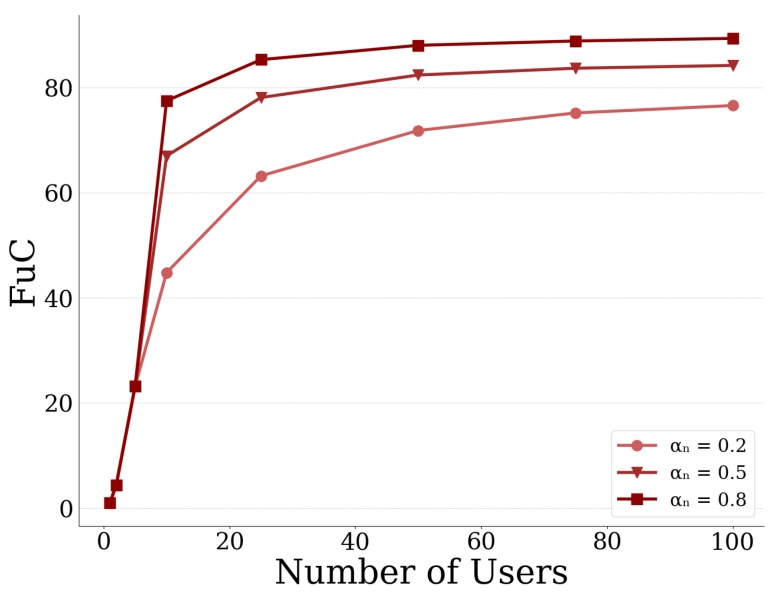
Fragility under Competition vs. no. of users for different sensitivity parameters $\alpha_{n}$.

**Figure 15:**
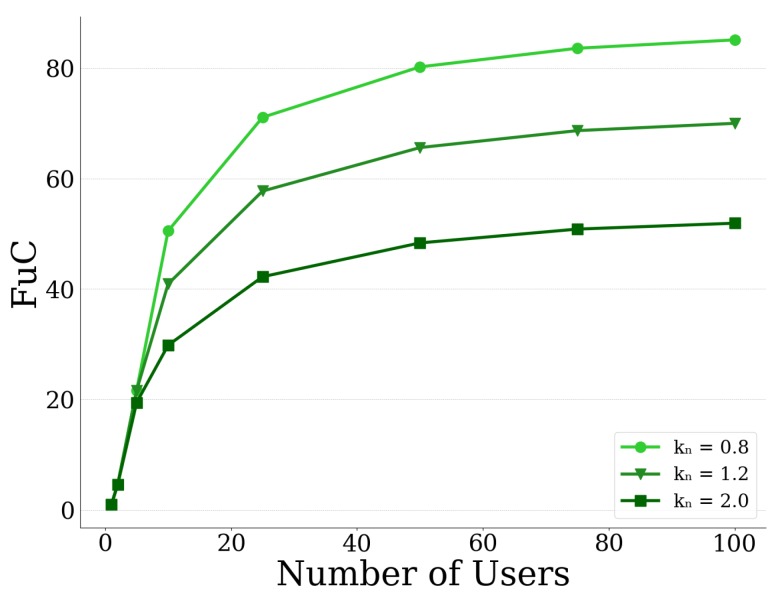
Fragility under Competition vs. no. of users for different loss aversion indices $k_{n}$.

In Figure 14 and Figure 15, we present the FuC metric as a function of the number of users in the system, for different values of the sensitivity parameter $\alpha_{n}$ and the loss aversion index $k_{n}$, respectively. In both figures, we observe that, as the number of users increases, the FuC increases as well, since more users are competing for the CPR and consequently more data are offloaded to the server, until it eventually plateaus. Concerning the effect that the prospect theoretic parameters have on the FuC metric, in Figure 14, we can see that the higher the value of the sensitivity parameter $\alpha_{n}$, the higher the FuC as well. This is justified by the fact that, the higher the values of $\alpha_{n}$, the greater the sensitivity of the users towards gains and losses of higher magnitude compared to those of smaller magnitude (Figure 12). As a result, users tend to offload more data to the MEC server and the server is more prone to failure, and accordingly an increase in FuC is expected. With respect now to the loss aversion index $k_{n}$, we can see in Figure 15 that, as $k_{n}$ increases, the FuC decreases. This is due to the fact that, as $k_{n}$ increases, users become more loss averse and thus they tend to offload less data to the MEC server in order to avoid potential failure as already shown in Figure 13. The less data are offloaded to the server, the less the probability that the server will fail, thus resulting in lower FuC. Furthermore, based on the results of Figure 14 and Figure 15, the FuC appears initially more sensitive to the number of users in the case $\alpha_{n}$ compared to $k_{n}$, our setting and experiments. It is clarified that the overall observed increasing trend of the FuC w.r.t. to the increasing number of users in these figures is well aligned with the fact that the failure probability is an increasing function of the total offloaded data of all users. However, the actual slope of the corresponding curves mainly depends on the used values for $\alpha_{n}$ and $k_{n}$ for the generation of these curves, which are selected here only for demonstration purposes, and are not correlated with each other in any way.

## 7. Conclusions

In this paper, a resource-based pricing and user risk-aware data offloading framework is proposed for UAV-assisted multi-access edge computing systems. In particular, a usage-based pricing mechanism is introduced regarding the exploitation of the MEC server’s computing capabilities by the users, and is properly incorporated within the principles and modeling of Prospect Theory, which is used to capture the users’ risk-aware behavior in the overall data offloading decision-making. Initially, the user’s prospect-theoretic utility function is formulated by quantifying the user’s risk seeking and loss aversion behavior, while taking into account the pricing mechanism. Accordingly, the users’ pricing and risk-aware data offloading problem is formulated as a distributed maximization problem of each user’s expected prospect-theoretic utility function and addressed as a non-cooperative game among the users. The existence of a Pure Nash Equilibrium for the formulated non-cooperative game is shown based on the theory of submodular games. An iterative and distributed algorithm is introduced that converges to the PNE, following the learning rule of the best response dynamics. Detailed numerical results are presented highlighting the operation feature and scalability properties of the proposed framework, while at the same time providing useful insights about the benefits of adopting the usage-based pricing scheme.

Our current and future research work focuses on treating the overall key problem of data offloading in various cloud computing environments, such as fog computing, where a large number of computing devices imposes additional scalability and stability challenges. Moreover, it is noted that, in this work, the data offloading problem was mainly treated from a computing resources perspective. However, depending on the environment assumed, the overall process could be affected by the wireless communication aspects between the UAV and users. The proposed framework could be adapted and extended to treat this aspect, either implicitly through the cost factors and functions considered when using the server resources, or explicitly by modeling the transmission characteristics (e.g., delay, rate, energy) involved in the offloading process.

## Figures and Tables

**Table 1:** Typical values for simulation parameters [36,37].

Parameter	Value	Description
$b_{n}$	$10^{7}\pm 10^{6}$ [bytes]	User’s *n* computation task’s input data
$d_{n}$	$8*10^{9}\pm 10^{9}$ [CPU cycles]	CPU cycles required to accomplish user’s *n* computation task
$f_{n}$	$6*10^{9}\pm 10^{9}$ [CPU cycles/sec]	User’s *n* device’s computational capability
$\gamma_{n}$	$4*10^{-9}\pm 10^{-9}$ [Joules/CPU cycles]	Coefficient of the locally consumed energy per CPU cycle
$a_{n}$	0.2	User’s *n* sensitivity on gains and losses
$k_{n}$	1.2	User’s *n* loss aversion parameter
$c_{n}$	$0.5*\frac{b_{n}}{d_{n}}\left(1-\frac{1}{{\hat{t}}_{n}{\hat{e}}_{n}}\right)$ [1/CPU cycles]	Pricing factor (satisfies condition of Theorem 1)
θ	$2*10^{-11}$	Parameter denoting the processing capability of the server, centers the sigmoid on to realistic values of offloading data

**Table 2:** Algorithm’s execution time per user for a different number of users.

N	Iterations	Time Per User [s]
1	3	0.0036
2	3	0.0042
5	3	0.0049
10	6	0.0095
25	14	0.0122
50	31	0.0282
75	54	0.0640
100	83	0.0979
